# Aldosterone-Producing Adrenal Oncocytoma: A Rare Cause of Primary Aldosteronism

**DOI:** 10.7759/cureus.88168

**Published:** 2025-07-17

**Authors:** Irida Kecaj, Ergita Nelaj, Ilir Gjermeni, Kei Xhixhabesi, Denis Godaj, Ina Refatllari, Irda Rrugeja

**Affiliations:** 1 Department of Internal Medicine, University Hospital Center "Mother Teresa", Tirana, ALB; 2 Department of Internal Medicine, Faculty of Medicine, University of Medicine, Tirana, ALB; 3 Department of Internal Medicine, University of Medicine, Tirana, ALB; 4 Department of Surgery, University Hospital Center "Mother Teresa", Tirana, ALB; 5 Department of Cardiology, University Hospital Center "Mother Teresa", Tirana, ALB; 6 Department of Pathology, University Hospital Center "Mother Teresa", Tirana, ALB

**Keywords:** adrenal incidentaloma, adrenocortical adenoma, adrenocortical oncocytoma, hypertension, laparoscopic adrenalectomy

## Abstract

Adrenal oncocytomas are rare, usually non-functional tumors that are often found incidentally. In some cases, however, they may present with endocrine hyperfunction, leading to diagnostic and therapeutic challenges.

We report the case of a 63-year-old male patient with a long-standing history of hypertension, managed with antihypertensive therapy. Due to persistently elevated blood pressure despite treatment, the patient underwent an evaluation for secondary causes of hypertension. Hormonal assessment demonstrated a high concentration of plasma aldosterone, with concurrently suppressed renin levels, resulting in a significantly raised aldosterone-to-renin ratio. Abdominal computed tomography (CT) identified a 1.5 cm solid lesion in the left adrenal gland, radiologically consistent with an adenoma. Subsequent histological examination confirmed a diagnosis of adrenal cortical adenoma composed predominantly of oncocytic cells.

This case illustrates an unusual presentation of a hormonally active adrenal oncocytoma manifesting as a microadenoma. Although oncocytic adrenal tumors are often large and non-secretory, our findings highlight that small lesions may also exhibit endocrine activity and be responsible for resistant hypertension. Accurate biochemical evaluation, timely imaging, and appropriate surgical intervention are essential for diagnosis and cure.

While histological criteria may support a benign nature, the presence of hormonal activity justifies clinical vigilance. This case underlines the importance of considering functional adrenal oncocytomas in the differential diagnosis of primary hyperaldosteronism, even when imaging reveals small tumors. It also reinforces the need for individualized patient follow-up planning, taking into account tumor behavior and biochemical activity.

## Introduction

Oncocytomas are epithelial tumors composed of large, round, or polygonal cells that contain eosinophilic cytoplasm due to an abundant accumulation of mitochondria. The granular texture of the cytoplasm reflects this excess mitochondrial content [1].

Oncocytomas are mainly benign tumors that usually originate from the thyroid, parathyroid, salivary, or pituitary glands [2]. The prevalence of adrenal incidentalomas in imaging studies is about 3% in adults >50 years old, and up to 10% in patients >80 years old; these include cortical adenomas, cysts, myelolipomas, ganglioneuromas, pheochromocytomas, adrenocortical carcinomas, or adrenal metastases [3]. Adrenal oncocytomas account for approximately 1.8% of adrenal incidentalomas, specifically in selected surgical series [4]. The literature shows that the finding of oncocytoma in the adrenal glands is extremely rare, with 287 cases identified so far; these are mainly non-functional tumors discovered by chance. Approximately 30% of adrenal oncocytomas display either malignant potential or evidence of hormonal hyperfunctionality [5]. The age of identification varied from 15 years to 77 years, with a prevalence 2.5 times higher in women and 3.5 times more frequent in the left adrenal gland [2].

Reported recurrence (8%) and mortality (3%) rates in adrenal oncocytomas generally refer to the full spectrum of oncocytic variants, including benign, borderline, and malignant forms. It is important to note that recurrence and mortality are predominantly associated with the malignant subtype, as most adrenal oncocytomas are benign and clinically indolent [1,2], emphasizing the importance of patient follow-up. Adrenal oncocytomas are usually large (>4-5 cm), suggestive of malignancy, and indistinguishable from adrenocortical carcinomas by imaging [1], highlighting the importance of the histopathological examination in diagnosis.

## Case presentation

A 63-year-old male was hospitalized in the emergency department for hypertensive encephalopathy, with high blood pressure levels resistant to medication. His medical history included arterial hypertension for 15 years and type 2 diabetes mellitus for 4 years. His current antihypertensive therapy was Olmesartan/Hydrochlorthiazide 40/12.5 mg daily, Lercanidipine 20 mg daily, and Moxonidine 1 mg daily, along with Metformin 1 g daily for his diabetes. He stated that in the last six months, he had frequent hypertensive crises for which he went to the emergency service. The results of routine blood tests, liver function, blood lipids, blood glucose, and thyroid function were normal, except for mildly low potassium levels (K+ 3.2 mmol/L).

After a cerebrovascular accident was ruled out with supraaortic CT, the patient underwent more detailed imaging examinations to assess target organ damage from hypertension. The echocardiography exam revealed severe left ventricular hypertrophy (septal/posterior wall thickness 17/14 mm) without left ventricular outflow tract (LVOT) obstruction. The left and right ventricles were of normal dimensions and function, as well as the atrium, with mild mitral regurgitation, while a pulmonary artery pressure (PAP) of 35 mmHg was measured. An abdominal ultrasound showed no pathological findings. The dilated fundoscopic exam demonstrated arterial narrowing with focal irregularities, compatible with stage II retinal damage.

Hyperaldosteronism was suspected due to resistant blood pressure and organ damage due to hypertension, including severe left ventricular hypertrophy, stage 2 hypertensive retinopathy, microalbuminuria, and hypokalemia. Antihypertensive therapy was changed to verapamil 240 mg/day and doxazosin 4 mg/day two weeks before measuring plasma renin activity (PRA), plasma aldosterone concentration (PAC), and the aldosterone/renin ratio (ARR). Serum aldosterone and renin levels were 59.3 ng/dL and 0.23 mIU/L, respectively, and the ARR was calculated to be 257. Laboratory-specific tests are summarized in Table 1.

**Table 1:** Specific laboratory data ALB, albumin; K+, potassium; T3, triiodothyronine; T4, serum thyroxine; TBIL, total bilirubin; TSH, thyroid-stimulating hormone; ARR, aldosterone/renin ratio

Laboratory assay	Value	Reference range
K^+ ^(mmol/L)	3.2	3.5 -5.1
Albuminuria 24h (mg/24h)	243	< 300
TSH (mU/L)	1.512	0.35-4.94
T_3 _(ng/ml)	3.27	1.58-3.91
T_4 _(mU/L)	1.13	0.7-1.48
Plasma renin activity (µIU/mL)	0.23	2.8–39.3
Plasma aldosterone concentration (ng/dL)	59.3	1.76-23.2
ARR	257	<2.4
ACTH (pg/mL)	12	7.2 – 63.3
Cortisol (ug/ml) 8 am	15.7	3.7-19.4
Cortisol (ug/ml) 16 am	10.2	2.9-17.3

**Figure 1:**
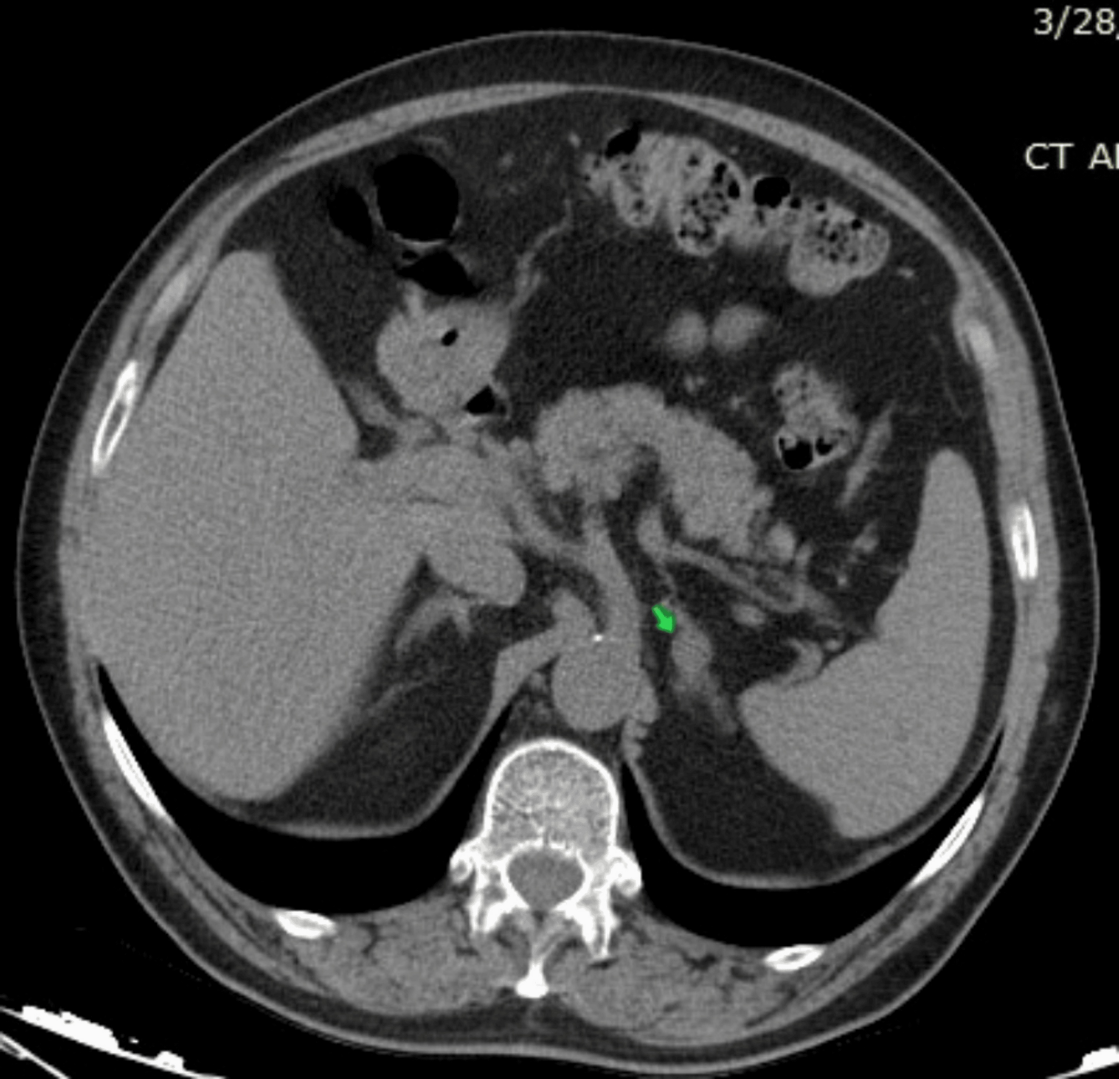
Computed tomography scan of the abdomen The arrow indicates the hypodense nodular formation in the left adrenal gland.

Since the hormone test suggested primary hyperaldosteronism, we decided to perform an imaging study of the adrenal glands. Adrenal CT identified a solid hypodense nodule in the left adrenal gland, which was 15×10 mm in size, consistent with adenoma (Figure 1).

**Figure 2:**
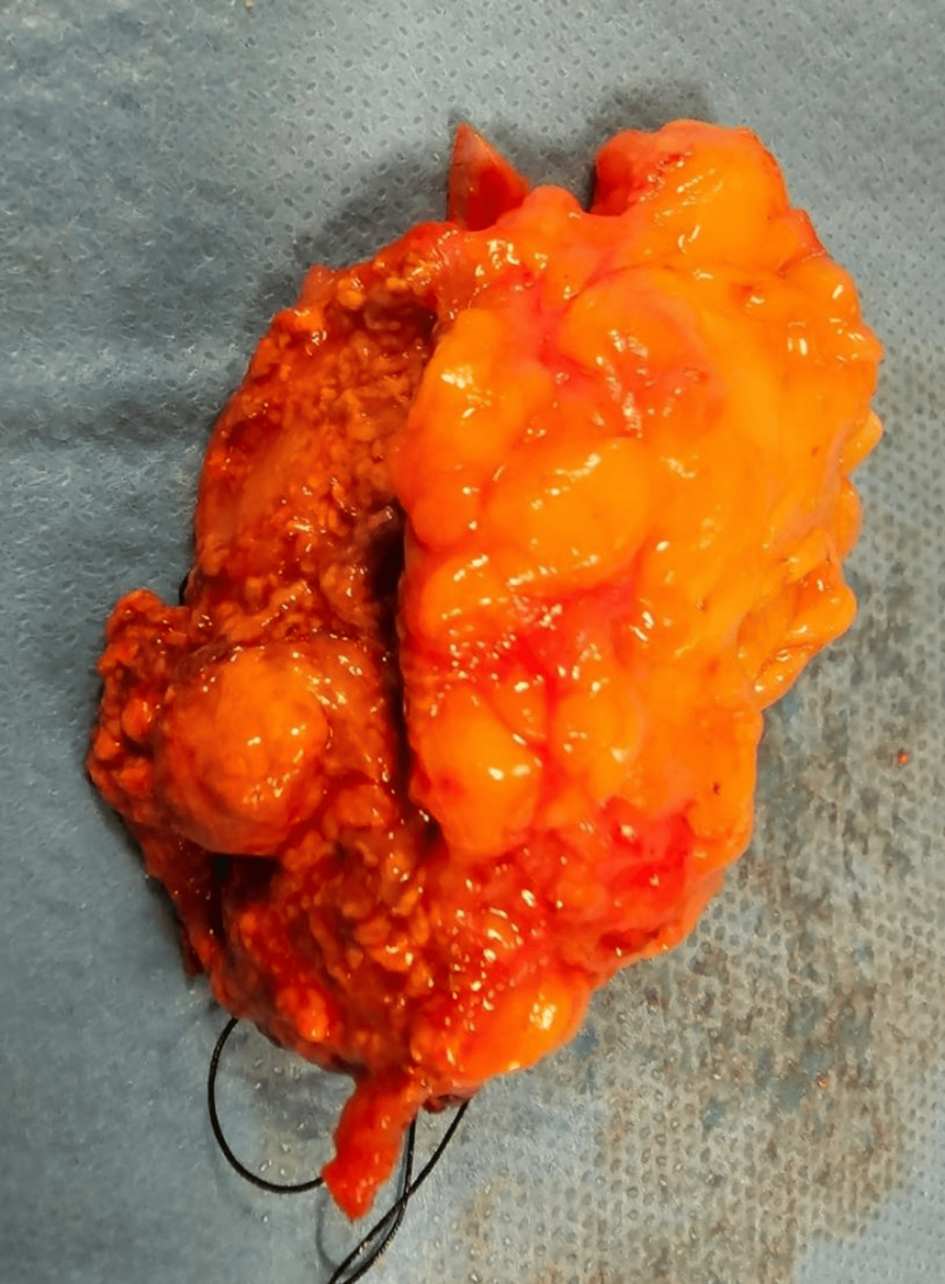
Surgical specimen after adrenalectomy

Since the diagnosis of primary hyperaldosteronism was confirmed by imaging examination in addition to hormonal analysis, it was decided that the patient should undergo laparoscopic surgery (Figure 2).

**Figure 3:**
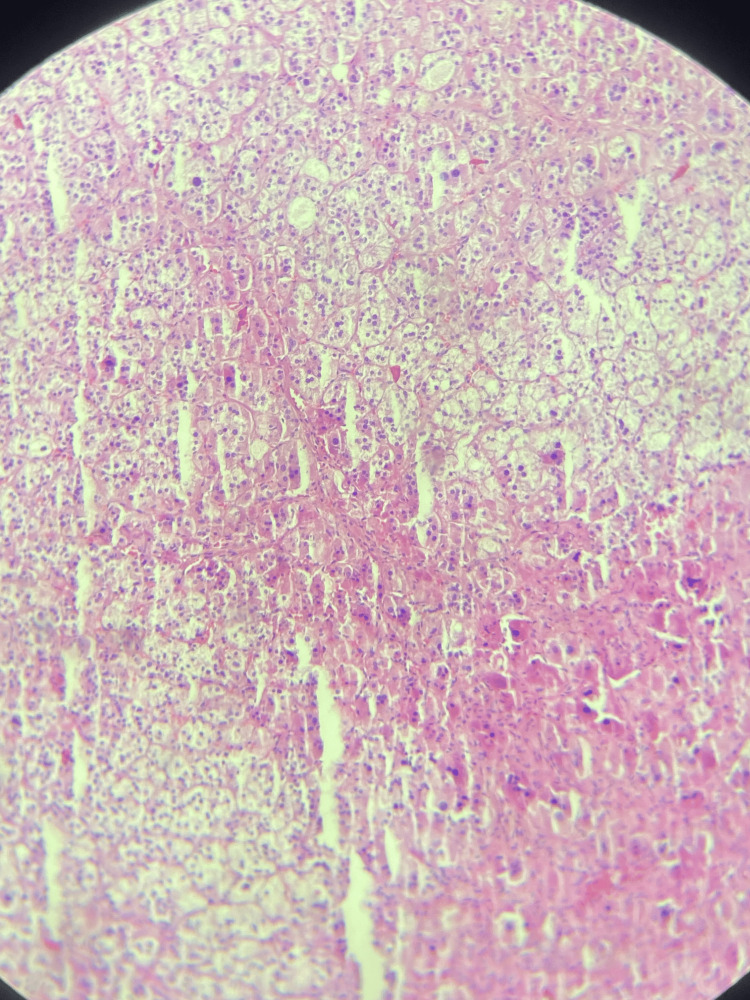
Cortical adenoma with oncocytic cells

The histopathological examination of the surgical specimens revealed a cortical adenoma with oncocytic cells (Figure 3).

Postoperatively, the patient’s blood pressure and serum potassium levels normalized without the need for mineralocorticoid receptor antagonists. At the three-month follow-up, he remained normotensive, with no clinical or biochemical evidence of recurrence, and his hormonal parameters were within normal limits. He is currently under close surveillance, with a scheduled six-month imaging follow-up and annual clinical and laboratory monitoring thereafter, in line with recommendations for functional adrenal tumors despite benign histology.

## Discussion

The group of extremely rare adrenocortical oncocytic neoplasms includes oncocytomas, oncocytic neoplasms of uncertain malignancy, and oncocytic carcinomas [6], with differential diagnosis including adrenal adenoma, gangliocytoma, gangliocytic paraganglioma, and lipoma [7]. Hormonally active adrenal oncocytomas reported in the literature mainly include Cushing's syndrome, pheochromocytoma, and hyperandrogenism [6,8]. Aldosterone-secreting adrenal oncocytoma is an extremely rare variant of adrenal oncocytic tumors, and, as we searched and read in the endless literature, there are very few cases of them. We found only seven cases over the years, predominantly in adults, where the masses were mostly over 4 cm, and only one of them was malignant, one was borderline, and the others were benign [6,8-12]. Although traditionally considered benign and nonfunctional tumors, recent data suggest that up to 20% of oncocytomas display some malignant elements and 10-20% are functional tumors [13]. It almost occurs in adults [14]; however, rare occurrences in pediatric patients have also been documented [10].

Our patient was considered to have essential hypertension, resistant to treatment. We point out the necessity of investigating secondary causes of hypertension, mainly in resistant forms [15]. Primary hyperaldosteronism (PA) is the most common endocrine cause of hypertension, with a characteristic triad of hypertension, metabolic alkalosis, and hypokalemia [16]. Traditionally, one of the diagnostic criteria for primary hyperaldosteronism was hypokalemia, but it was later found that only 9-37% of patients with aldosterone overproduction have hypokalemia [17]. Although hypokalemia is not a diagnostic criterion for PA, the European Society of Endocrinology recommends screening for PA in patients with hypertension and hypokalemia [16]. 

Since our patient presented with resistant blood pressure values ​​and hypertension-mediated organ damage, such as severe left ventricular hypertrophy with diastolic dysfunction, grade II hypertensive retinopathy, and microalbuminuria, it was decided to examine for PA. The renin/aldosterone ratio was very high, after their dosing after more than two weeks, referring to the recommendations for stopping preparations that interfere with the renin-angiotensin-aldosterone system, at least two weeks before hormonal dosing [16]. According to the European Society of Endocrinology Guidelines, there is no need to perform confirmatory tests in patients with clear hormonal tests for PA, such as positive ARR, high PAC (>20 ng/dL), or hypokalemia, testing that was omitted in our patient and proceeded with imaging examination of the adrenal gland with a CT scan [16]. Genetic testing is recommended for familial forms of hyperaldosteronism, when PA is identified in patients <20 years of age, there is a family history of PA or history of stroke in an individual <40 years of age [18], which is not compatible with the characteristics of our patient. Since the adrenal scan showed a 1.5 cm nodule in the left adrenal gland, consistent with adenoma, and the impossibility of performing adrenal venous sampling in our country, considering the patients' preferences, it was decided to perform laparoscopic adrenalectomy. Histological examination of the adrenal mass revealed cortical adenomas with oncocytic cells.

Distinguishing between malignant and benign forms of adrenal oncocytoma is a pathological and imaging challenge. Weiss was the first to propose a histologic scoring system for adrenal tumors [19]. After that, Bisceglia recommended the pathological criteria used to distinguish between benign and malignant forms, where the presence of at least one of the major criteria (nuclear pleomorphism, a mitotic rate > 5 per 50 high-power fields, and venous invasion) is indicative of malignancy [20]. The presence of at least one of the minor criteria (tumor size over 10 cm or weight over 200 g, necrosis, sinusoidal invasion, and capsular invasion) is indicative of tumors of undetermined malignancy. The absence of criteria, whether major or minor, is an indicator of benignity [21], as in our case. Regarding prognosis, as reported by Kanitra et al. in a recent literature review, the 5-year overall survival rates were 100% for benign tumors, 88% for borderline tumors, and 47% for malignant tumors [22].

Radiological features that raise suspicion for malignancy include tumor size greater than 5 cm, irregular shape, and unclear and inhomogeneous margins [23]. Although our case exhibited imaging features characteristic of a benign adrenal adenoma and fulfilled the Lin-Weiss-Bisceglia criteria for a non-malignant oncocytic neoplasm - criteria that typically do not mandate routine follow-up - we recommend continued clinical and biochemical surveillance. This recommendation is based on the tumor’s functional nature and the rare but documented risk of recurrence, even in histologically benign lesions [22]. The patient experienced an uneventful postoperative course, with normalization of blood pressure and potassium levels, and no further need for mineralocorticoid receptor antagonists. At three months, clinical and laboratory follow-up showed no signs of recurrence or residual disease, supporting the tumor’s benign behavior. Nevertheless, given the endocrine activity of the lesion and the potential for late recurrence, we advise annual monitoring with clinical assessment, hormonal evaluation, and periodic imaging.

For adrenal masses, laparoscopic surgery is the treatment of choice, and it has advantages over open surgery, such as less postoperative pain, reduced hospital stays, reduced ileus, and better cosmetic appearance. This procedure appears safe for benign lesions up to 12 cm [24].

## Conclusions

This case highlights the importance of considering adrenal oncocytomas in the differential diagnosis of adrenal adenomas, even when they present as small lesions. While oncocytic adrenal tumors are typically large and nonfunctional, our findings underscore that hormonally active microadenomas with oncocytic features can occur and should not be overlooked during evaluation. According to the major and minor criteria of the Lin-Weiss-Bisceglia score, these tumors are classified as benign, borderline, or malignant. Laparoscopic adrenalectomy is the most common treatment method after excluding local invasion in preliminary imaging examinations. Given the hormonal activity and the rare but documented risk of recurrence, periodic clinical and biochemical monitoring remains essential even in cases classified as benign.
